# Comparative Effects of Clump-Based and Traditional Selective Harvesting on Understory Biodiversity in Sympodial Bamboo Forests

**DOI:** 10.3390/plants14162578

**Published:** 2025-08-19

**Authors:** Ying Zhang, Chaohang Zhang, Zuming Wang, Haoting Li, Haofeng Bao, Fengying Guan, Chaomao Hui, Weiyi Liu

**Affiliations:** 1 College of Forestry, Southwest Forestry University, Kunming 650224, China; yingz@swfu.edu.cn (Y.Z.); zch20010413@163.com (C.Z.); lighearting@foxmail.com (Z.W.); haoting1020@163.com (H.L.); bhfzmx@163.com (H.B.); 2 International Center for Bamboo and Rattan, Beijing 100102, China; guanfy@icbr.ac.cn

**Keywords:** *D. giganteus*, clump-based harvesting, species diversity, soil chemical properties

## Abstract

To improve the efficiency and reduce the cost of traditional sympodial bamboo harvesting, this study evaluated the effects of four harvesting intensities—selective harvesting, one-third clump, one-half clump, and complete clump harvesting—on understory plant diversity in pure *Dendrocalamus giganteus* stands over a five-year recovery period. A total of 36 species were recorded in the first year, increasing to 71 in the third year and stabilizing at 74 species by year five. Understory α-diversity showed an increasing trend followed by a decline. In the early recovery stage, species diversity was significantly correlated with soil chemical properties (*p* < 0.05), but no significant correlation was observed in the later stage. Fuzzy membership function analysis indicated that the 1/2 clump harvesting treatment outperformed others, ranking as follows: 1/2 clump > 1/3 clump > selective > complete clump harvesting. These results suggest that 1/2 clump harvesting is optimal for promoting understory vegetation growth, but its positive effects on biodiversity are time-limited, with the plant community showing a trend toward simplification over time.

## 1. Introduction

Harvesting is a common disturbance method in forest ecosystems and is a management practice implemented for the purpose of timber production [1,2]. By reducing forest stand density and altering spatial structure, as well as adjusting the number and functional composition of retained trees [3], it aims to improve stand productivity, enhance wood quality, and cultivate large-diameter timber [4]. Selective harvesting, as a traditional harvesting method, is considered ecologically beneficial as it maintains forest structure, protects biodiversity, improves wood quality, and supports sustainability. In recent years, rising labor costs have significantly impacted the economic benefits of bamboo forests. Labor expenses have increasingly become a major constraint on the industrial development of bamboo forestry. Diversified forest management practices have gradually been introduced into bamboo forest operations. For example, strip harvesting methods designed to suit the growth characteristics of sympodial bamboo have been proposed. A width of 8 m was identified as the optimal strip width from the perspectives of economic and ecological value. This strip harvesting method significantly improves harvesting efficiency, reduces management costs, and enhances bamboo productivity [5,6,7,8,9,10,11]. Clumping bamboo, such as *D. giganteus*, differs from monopodial bamboo species like *Phyllostachys edulis* and *Neosinocalamus affinis*. Its traditional harvesting approach involves selective cutting, removing bamboo culms older than four years that have timber value. However, this approach is operationally challenging, inefficient in productivity, high in cost, and relies on low-level mechanization with limited operations. To address these issues, a mechanized harvesting method tailored to the growth pattern of clumping bamboo—referred to as clump-based harvesting—has been designed. After harvesting, bamboo stand density decreases, forming large canopy gaps, which inevitably causes substantial disturbance to the bamboo forest ecosystem [12]. While some studies have examined fertilizer regimes, bamboo growth, microclimate conditions, and short-term changes in biodiversity following clump-based harvesting [13,14,15,16], the long-term effects on understory plant diversity remain poorly understood.

Plant species diversity, as a fundamental feature of ecosystems, reflects species richness, evenness, and the relationship between community structure and its surrounding environment. It is essential for maintaining ecosystem functionality and structural stability [17,18,19,20]. The spatial distribution of plant diversity is significantly influenced by environmental factors. Diversity patterns determine the complexity of biological community structures and also reflect the types, organizational levels, and stability of plant communities, as well as their expression across habitats [4]. The formation of species diversity is influenced by various complex factors. Among them, the intensity and frequency of harvesting disturbances are key. Different harvesting intensities affect the survival and development of plant communities in distinct ways. Moderate disturbance does not necessarily reduce species richness and may even promote biodiversity [21,22,23]. Soil and plants interact in a complex and coordinated manner. Soil factors regulate plant growth and community composition, while plants improve soil properties through litter and root exudates [24,25,26]. By integrating diversity indices and soil factors, we can better understand plant community integrity and ecosystem functional stability [13,27].

*D.s giganteus* is a large, high-quality clumping bamboo species with tall, straight, and thick culms. It grows rapidly, possesses excellent mechanical properties, and is widely used in construction, handicrafts, and interior design. Its edible shoots can be processed into bamboo strips and dried products [28,29,30,31]. In this study, *D. giganteus* was selected as the research subject. Through plant community surveys, we analyzed vegetation recovery following clump-based harvesting, compared changes in understory species composition, α-diversity indices, and regeneration ability under different harvesting intensities over time, and identified the influencing factors on understory plant diversity. This study aimed to clarify the extent and temporal impact of ecological disturbance under different clump-based harvesting intensities, providing insights into two key questions: (1) What are the effects of varying harvesting intensities on understory plant diversity and how do they change over time? (2) How do stand characteristics relate to understory plant diversity and soil chemical properties, and what is the optimal clump-based harvesting intensity for clumping bamboo?

## 2. Materials and Methods

### 2.1. Study Area and Experimental Design

**Figure 1:**
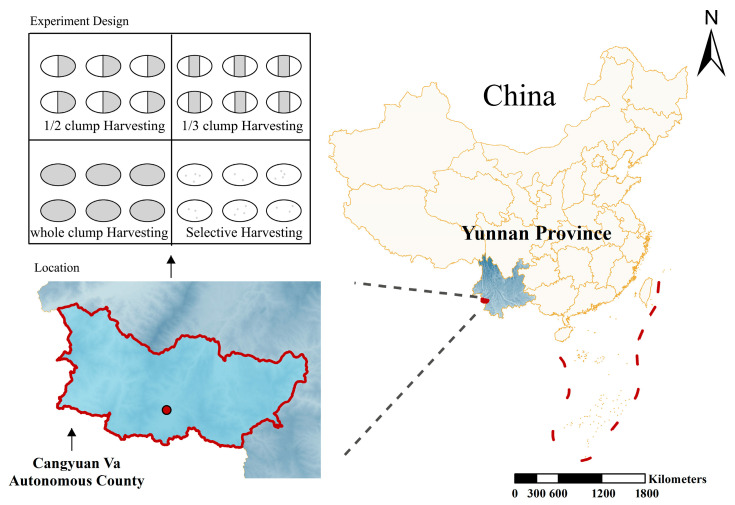
Distribution of the four harvesting intensity treatments in Cangyuan Va Autonomous County, Lincang City, Yunnan Province, China.

The study area is located in Menggan Village, Mengjiao Township, Cangyuan Va Autonomous County, Lincang City, Yunnan Province, China (geographic coordinates: 99°14′–99°15′ E, 23°11′ N), as shown in Figure 1. The elevation of the area ranges from 1330 m to 1450 m, with slopes between 21° and 28°, facing southeast. The region experiences a South Asian subtropical monsoon climate characterized by distinct seasons and a clear alternation between dry and wet periods. The average annual temperature is 17.4 °C, with extreme temperatures ranging from a minimum of −4.3 °C to a maximum of 33.7 °C. The annual precipitation averages 1763.5 mm, with approximately 1876.7 h of sunshine per year. The frost-free period lasts about 317 days annually. The soil in the study area is classified as Haplic Ferralsols, with a depth exceeding 80 cm and moderate-to-high fertility, providing favorable ecological conditions for the growth of *D*. *giganteus*.

In November 2018, a 6.67-hectare demonstration forest for *D*. *giganteus* harvesting and cultivation trials was established. The harvesting operations were completed in December 2018. As of December 2023, the recovery period had reached five years. The experiment included four harvesting intensity treatments: selective harvesting (A), half-clump harvesting (B), one-third clump harvesting (C), and whole-clump harvesting (D). Four experimental blocks were established: blocks A, B, and C each measured approximately 180 m × 20 m and contained nine standard plots (20 m × 20 m each). Block D measured approximately 360 m × 20 m and contained 18 standard plots (20 m × 20 m each). The site conditions and stand characteristics of the experimental plots are summarized in Table 1.

### 2.2. Understory Vegetation Survey and Soil Sample Collection

In early August 2024, three 5 m × 5 m shrub quadrats were randomly established within each standard plot. At the center of each shrub quadrat, a 1 m × 1 m herbaceous subplot was placed to record species composition, number of individuals (or clumps), plant height, and canopy cover for both shrubs and herbs. Soil samples were collected using a five-point (quincunx) sampling method, in which five random points were selected around each bamboo clump. As *D. giganteus* is a sympodial bamboo species and harvested clumps are retained in the field, the central-line sampling method was not applicable. Instead, the five-point method was consistently used. For each of the four harvesting treatments, three bamboo clumps were selected, and soil samples (~1 kg) were collected from three depth layers: 0–20 cm, 20–40 cm, and 40–60 cm. Surface litter was removed prior to homogenizing the samples, which were then air-dried at room temperature and stored for subsequent chemical analysis.

### 2.3. Determination of Soil Chemical Properties

Soil pH was measured using the potentiometric method with a soil-to-water ratio of 2.5:1. Soil organic matter was determined using the external heating potassium dichromate oxidation method. Total nitrogen was measured using the Kjeldahl method, and alkali-hydrolyzable nitrogen was determined by the diffusion method. Total phosphorus and available phosphorus were analyzed using the molybdenum-antimony anti-colorimetry method. Total potassium and available potassium were determined using a flame photometer [32].

### 2.4. Data Calculation

The importance value, a synthetic metric reflecting the relative dominance of species within a community, is a key indicator for assessing community structure [33]. The Shannon index quantifies the uncertainty of information; higher uncertainty corresponds to greater species diversity. Although the Simpson index showed relatively minor overall differences across harvesting treatments and recovery years, significant differences were still observed in certain treatments (*p* < 0.05). The Pielou evenness index reflects both the variation in species richness and the uniformity of species distribution within the community [20].

Based on the survey data, the important value (IV) of each species in the shrub and herbaceous layers and the α-diversity indices were calculated using the following formulas [34]:

Importance Value (IV)

(1) $$IV=\frac{\left(Relative\;Abundance+Relative\;Frequency+Relative\;Coverage\right)}{3}$$

Simpson Diversity Index (D)

(2) $$D=1-\sum_{i-1}^{N}{\left(P_{i}\right)}^{2}$$

Shannon–Wiener Diversity Index (H)

(3) $$H=-\sum_{i-1}^{N}\left(P_{i}\times {lnP}_{i}\right)$$

Margalef Richness Index (R)

(4) $$R=\frac{S-1}{ln\left(N\right)}$$

Pielou’s Evenness Index (J)

(5) $$J=\frac{H}{ln\left(S\right)}$$

where $P_{i}$ represents the relative abundance of species _*i*_ within a plot, defined as $P_{i}=\frac{n_{i}}{N}$, with $n_{i}$ being the number of individuals of species _*i*_, and *N* being the total number of individuals of all species in the plot. *S* denotes the total number of species present in the plot.

As shown in Table 2, the diameter at breast height (DBH) of *D. giganteus* under different treatments was measured, and the corresponding age class regression models for aboveground biomass of *D. giganteus* plantations were used to estimate the aboveground biomass per plant (kg/plant) based on the measured DBH values [35].

### 2.5. Data Analysis

One-way analysis of variance (ANOVA) was conducted to assess differences in species diversity among the four harvesting intensity treatments. The assumptions of normality and homogeneity of variances were tested using the Shapiro–Wilk and Levene’s tests, respectively. Pairwise comparisons of treatment means were performed using the least significant difference (LSD) test, with statistical significance set at *p* < 0.05. Principal component analysis (PCA) was employed to examine the relationships between species diversity and soil characteristics. All statistical analyses were conducted using SPSS 27.0. Data management was carried out in Excel 2016, and graphical visualizations were produced using Origin 2021.

## 3. Results

### 3.1. Species Composition and Importance Value of Understory Vegetation Under Different Harvesting Intensities

**Figure 2:**
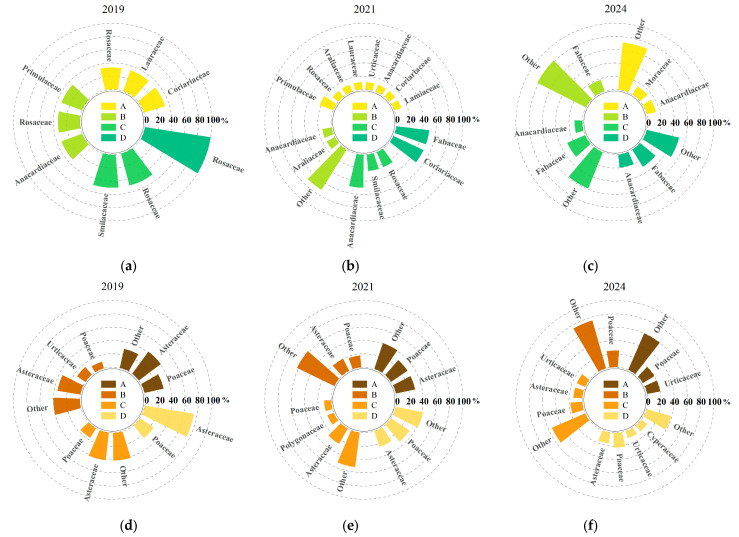
Dominant understory plant species composition under different harvesting intensities. Letters A, B, C, and D represent the four treatments, while (**a**–**c**) indicate shrub species composition, and (**d**–**f**) indicate herbaceous species composition.

According to the composition of major understory plant species under different harvesting intensities (Figure 2), both the number and composition of understory plant species changed significantly over time following clump harvesting. For ease of statistical analysis, species with a relative abundance less than or equal to 10% were grouped as “others.” In 2019 (the first year after harvesting), a total of 36 plant species were identified in the understory, belonging to 20 families and 34 genera. Among them, the shrub layer comprised six families, six genera, and six species, while the herbaceous layer comprised 14 families, 27 genera, and 30 species. By 2021 (the third year after harvesting), the number of species had increased significantly, with 71 plant species recorded, belonging to 37 families and 65 genera. Compared to 2019, the number of shrub species increased by 6, 11, 2, and 1 under selective harvesting, 1/2 clump harvesting, 1/3 clump harvesting, and complete clump harvesting, respectively. The total number of shrub species increased to 13 genera in 11 families. For the herbaceous layer, the number of species increased by 10, 20, 7, and 7, representing a 66.67% increase compared to 2019. In 2024 (the fifth year after harvesting), the number of understory species further increased to 74 species, belonging to 39 families and 67 genera, mainly from Fabaceae, Poaceae, Asteraceae, and Urticaceae, indicating a high level of species richness and relatively even family-level composition. Compared to 2021, the number of shrub species under each treatment continued to increase by 5, 8, 13, and 8; the number of herbaceous species increased by 17, 16, 3, and 6. Overall, the total number of species increased by 4.23% compared to 2021, although the growth rate slowed. In terms of growth trends by life form, the number of species in both shrub and herbaceous layers continued to increase with the recovery time, and the proportion of herbaceous species also showed an upward trend, indicating that harvesting disturbance to some extent promoted the recovery and reconstruction of understory plant diversity.

**Figure 3:**
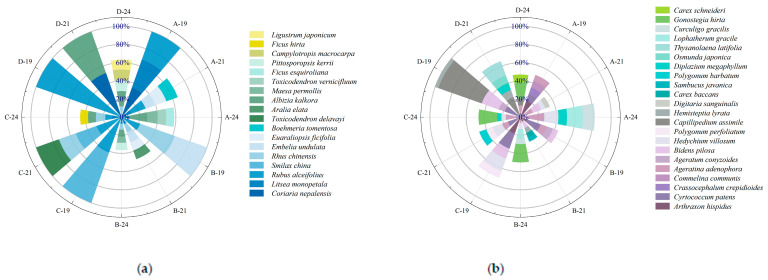
(**a**) Importance values of dominant shrub species; (**b**) importance values of dominant herbaceous species under different harvesting intensities. Letters A, B, C, and D represent the four harvesting treatments; numbers 19, 21, and 24 indicate the years 2019, 2021, and 2024, respectively.

Figure 3 illustrates the temporal dynamics of importance values for shrub and herbaceous species under varying harvesting intensities. Overall, species composition and dominance shifted substantially with restoration time following harvesting interventions. In 2019, six shrub species were recorded across treatments. *Rubus alceifolius* was present in all plots and exhibited pronounced dominance, with importance values of 32.27%, 21.99%, 41.67%, and 100% under selective, 1/2 clump, 1/3 clump, and complete harvesting, respectively, especially absolute in the complete harvesting plots. In the herbaceous layer, *Arthraxon hispidus* and *Cyrtococcum patens* were predominant in the selective, 1/2, and 1/3 clump treatments, whereas *Capillipedium assimile* and *Bidens pilosa* dominated under complete harvesting. By 2021, shrub species richness increased, and dominant species shifted: selective harvesting: *Coriaria nepalensis*, *Litsea monopetala*, and *R. alceifolius* dropped to 8.26%, 11.62%, and 16.81%, respectively. In the 1/2 clump treatment: *Rhus chinensis*, *Embelia undulata*, and *R. alceifolius* declined to 9.52%, 11.48%, and 7.82%. In the 1/3 clump treatment: *Toxicodendron delavayi* and *R. chinensis* emerged as dominants at 26.67% and 19.63%. Complete harvesting: *R. alceifolius* was replaced by *C. nepalensis* and *Albizia kalkora* at 51.79% and 48.21%, respectively. In the herb layer, species distribution became more even: selective harvesting: *Ageratina adenophora* increased to 9.22%, while *A. hispidus* and *C. patens* declined to 4.1% and 5.45%. In the 1/2 clump treatment: *C. patens* and *A. hispidus* declined, while *B. pilosa* rose to 7.74%. In the 1/3 clump treatment: *C. patens*, *Hedychium villosum*, and *Polygonum perfoliatum* reached 15%, 10.96%, and 7.52%, respectively. Complete harvesting: *C. assimile* and *B. pilosa* dropped to 23.69% and 8.22%. By 2024, community structure became increasingly complex: in shrub layers, *A. kalkora* dominated under selective harvesting; *L. monopetala* declined to 8.91%. In the 1/2 and 1/3 clump treatments, *L. monopetala*, *R. chinensis*, and *R. alceifolius* showed reduced importance. New shrub species were more prevalent in complete harvesting plots, though *A. kalkora*’s dominance fell to 16.74%, indicating diversification. In the herbaceous layer: under selective harvesting, species richness declined, with *A. adenophora* remaining dominant and *H. villosum* increasing to 15.58%. In the 1/2, 1/3, and complete treatments, *Gonostegia hirta* emerged as the dominant species, with *A. adenophora* maintaining moderate dominance (6.62–12.19%). These findings suggest that harvesting intensity and recovery time significantly influence the composition and hierarchical structure of understory vegetation.

### 3.2. Changes in Understory Plant Diversity Indices Under Different Harvesting Intensities

**Figure 4:**
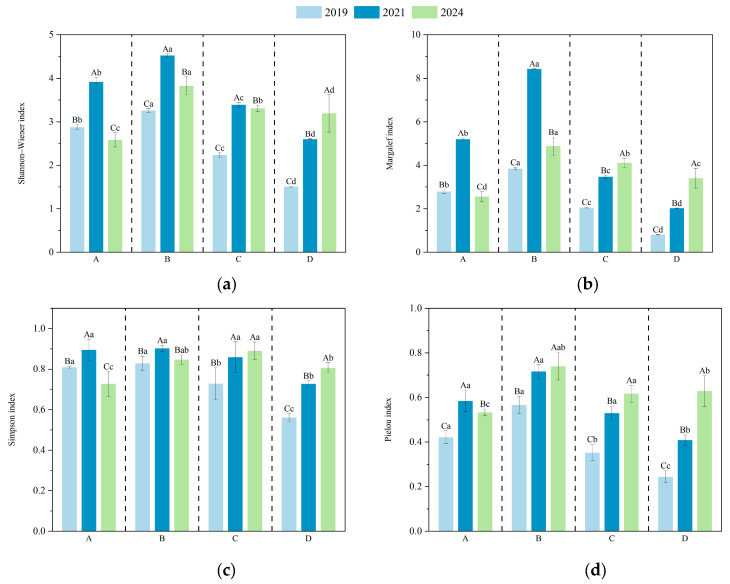
Species diversity indices of understory vegetation under different harvesting intensities. Lowercase letters indicate significant differences (*p* < 0.05) among treatments within the same year, while uppercase letters indicate significant differences (*p* < 0.05) among years within the same treatment. (**a**) Shannon indexa; (**b**) Margalef index; (**c**) Simpson index; (**d**) Pielou index.

The diversity indices of understory vegetation in plots with different harvesting intensities are shown in Figure 4. ANOVA results indicated that clustered harvesting had a significant effect on the Shannon index of understory species in *D. giganteus* forests. In the first and third years after harvesting, the Shannon index increased initially and then declined with increasing disturbance intensity, with the highest value observed in the 1/2-clump harvesting treatment and the lowest in the full clump harvesting treatment. By the fifth year, the Shannon index declined in the selective harvesting, 1/2 clump, and 1/3 clump treatments, while it increased in the full clump treatment. This suggests that the 1/2 clump harvesting treatment promotes a favorable competitive structure and is most effective in enhancing community diversity.

As the recovery period progressed, the Margalef index in the full clump harvesting treatment significantly increased from the first to the third year compared with the selective, 1/2 clump, and 1/3 clump treatments. By the fifth year, the differences in Margalef index among treatments were significant: the 1/3 clump and full clump treatments showed increasing trends, while the selective and 1/2 clump treatments exhibited declines, though values remained higher than in 2019. These results indicate that moderate-intensity harvesting is conducive to increasing species richness. With the increasing canopy closure, the diversity of each treatment tended to return to pre-harvest levels.

In the selective (A) and full clump harvesting (D) treatments, Simpson index values significantly differed across years, indicating that community structure was still fluctuating. In contrast, no significant changes were observed between 2021 and 2024 in the 1/2 clump (B) and 1/3 clump (C) treatments, suggesting that community structure under these treatments was relatively stable. Among all treatments, the Simpson index for the B treatment remained consistently high, peaking in 2021, indicating strong community evenness and stability. By 2024, the Simpson index ranked as follows: 1/2 clump > 1/3 clump > selective > full clump harvesting. This suggests that the 1/2 clump treatment helps promote a more balanced distribution of species dominance, while both 1/3 clump and full clump treatments may reduce community diversity. Although selective harvesting involves lower disturbance, it showed relatively poor recovery of understory species diversity.

The ANOVA results showed that by the fifth year of recovery, the Pielou index in the selective harvesting treatment significantly differed from that in the 1/2 clump, 1/3 clump, and full clump treatments, with the highest value observed in the 1/2 clump treatment. This indicates that the 1/2 clump harvesting is most favorable for promoting species coexistence within the community. In contrast, both excessive and insufficient disturbance can lead to the dominance of a few species, thereby reducing community evenness.

### 3.3. Principal Component Analysis of Diversity Indices, Stand Structure, and Soil Chemical Properties

**Figure 5:**
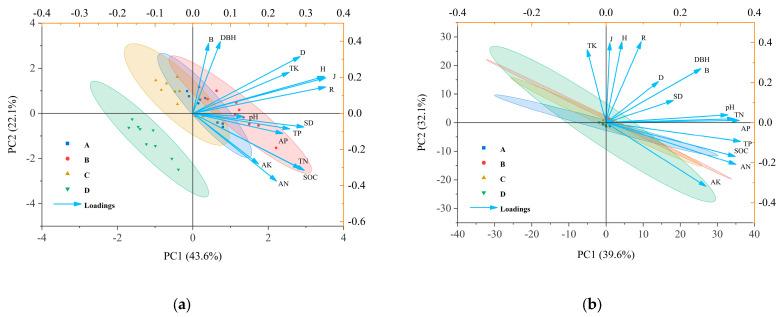
(**a**) shows the principal component analysis (PCA) of stand characteristics, diversity indices, and soil chemical properties at the early stage of harvesting in 2019; (**b**) shows the PCA of stand characteristics, diversity indices, and soil chemical properties after five years of recovery in 2024. Index: SOC: soil organic carbon; TN: total nitrogen; TP: total phosphorous; TK: total potassium; AN: alkali hydrolyzable nitrogen; AP: available phosphorous; AK: available potassium; H: Shannon–Wiener index; D: Simpson index; J: Pielou index; R: Margalef index; DBH: diameter at breast height; SD: stand density; B: biomass of individual plant.

As shown in Figure 5, the first two principal components in 2019—PCA1 and PCA2—accounted for 43.6% and 22.1% of the total variation, respectively. One year following harvesting recovery, soil pH was significantly positively correlated with stand density (SD). Soil organic carbon (SOC) exhibited extremely significant positive correlations with SD, several diversity indices including the Shannon–Wiener index (H), Margalef index (R), and Pielou index (J), as well as with total phosphorus (TP), total nitrogen (TN), available potassium (AK), available phosphorus (AP), and available nitrogen (AN). Similarly, TN was strongly positively associated with SD, the same diversity indices (H, R, J), and TP. TP showed robust positive correlations with SD, H, R, J, and total potassium (TK). TK was also positively and significantly related to SD and all four diversity indices: Simpson index (D), H, R, and J.

In 2024, the first two principal components, PCA1 and PCA2, accounted for 39.2% and 33.2% of the total variation, respectively. As illustrated in Figure 5b, no statistically significant correlation (*p* > 0.05) was observed between soil chemical properties and plant species diversity in the *D*. *giganteus* forest after five years of post-harvest recovery.

### 3.4. Effects of Harvesting Intensity and Recovery Time on Understory Plant Diversity

As shown in Table 3, both harvesting intensity, recovery time, and their interaction had highly significant effects (*p* < 0.01) on all four diversity indices: the Shannon–Wiener index, Simpson index, Pielou evenness index, and Margalef richness index. Among them, the Margalef index was most sensitive to harvesting intensity, while the Simpson index showed the least response across all factors. Overall, the influence of both main effects and their interaction followed the order of: Margalef richness > Shannon–Wiener > Pielou evenness > Simpson, indicating that species richness was more responsive to disturbance regimes than community evenness.

### 3.5. Evaluation of Diversity Indices, Stand Characteristics, and Soil Chemical Properties Based on Fuzzy Membership Function Analysis

Based on the results from two years of measurements on diversity indices, stand structure, and soil chemical properties, a fuzzy membership function analysis was performed. As shown in Table 4, the average membership values across all indicators followed the order: 1/2 clump harvesting > 1/3 clump harvesting > selective harvesting > complete clump harvesting. The 1/2 clump harvesting treatment consistently outperformed the others, indicating that this harvesting strategy is the most effective in promoting the growth of *D. giganteus* s stands.

## 4. Discussion

### 4.1. Effects of Different Clump Harvesting Patterns on the Understory Plant Community Structure of D. giganteus Forests

Stand density is a key factor influencing understory vegetation diversity [36]. In *D. giganteus*-dominated forests with a simplified overstory, the understory plays a pivotal role in maintaining biodiversity. The dense bamboo canopy limits light penetration and intensifies competition for resources, restricting the growth of understory plants. Clump harvesting creates substantial canopy gaps, thereby reshaping the spatial conditions for understory development [37]. Our study observed an increasing trend in the number of shrub and herbaceous species during the first three years of post-harvest recovery across all treatments. By the fifth year, species composition had shifted—particularly under selective harvesting, where dominance transitioned from herbaceous to shrub species, accompanied by a decline in species richness. In contrast, species numbers in the 1/2 and 1/3 harvesting treatments continued to grow, albeit slowly. These findings suggest that harvesting significantly enhances understory species diversity in the short term [38,39]. During the first year of recovery, herbaceous species predominated under all four treatments, with significantly higher species richness observed under selective, 1/2, and 1/3 harvesting compared to clear cutting. The highest richness occurred under the 1/2 harvesting treatment, then declined with increased intensity, reaching the lowest level in the clear-cut plots. This aligns with findings by Liu Size et al. [40], who noted that shrub richness in *Pinus massoniana* plantations responded more slowly to thinning than herbaceous species, likely due to shrubs’ need for more open space. By year three, species composition showed similar trends to year one, with some shifts in dominance, though Poaceae and Asteraceae families remained prevalent. By year five, species richness across treatments had stabilized, though the growth rate had slowed, and dominant species had been replaced. These patterns suggest that clump harvesting temporarily improves light and thermal conditions, accelerating the development of the herbaceous and shrub layers. However, as stand density increases over time and the canopy closes, light availability diminishes, reducing the competitiveness of understory species and potentially leading to species loss. These temporal dynamics contribute to the observed shifts in understory community structure in *D. giganteus* forests. Plant community succession driven by canopy gap dynamics has been widely reported across different forest ecosystems. In *D. giganteus* monocultures, the regulatory role of clump harvesting intensity in shaping the rate and trajectory of community recovery appears to exhibit a certain degree of generality. The structural evolution of communities under clump harvesting not only informs management strategies for tropical bamboo forests but also offers a valuable case for understanding forest community responses to disturbances across varying spatial scales [41].

### 4.2. Effects of Different Clump Harvesting Patterns on Understory Plant Diversity in D. giganteus Forests

The impact of harvesting disturbance on species diversity depends on disturbance characteristics such as intensity and frequency. Clump harvesting modifies the microenvironment by changing key local habitat factors such as light availability, temperature, moisture, and soil nutrient content. These changes increase spatial heterogeneity in microclimatic conditions, which in turn influence the structure and distribution of plant communities. These environmental changes ultimately shape the distribution patterns of plant communities and biodiversity [42,43,44]. In this study, we analyzed the effects of different clump harvesting intensities on understory plant diversity over a five-year period. The results indicated that the open spaces formed after clump harvesting created niches for other species to colonize the understory. Harvesting intensity significantly affected understory species diversity indices (*p* < 0.05). Among all treatments, the 1/2 clump harvesting exhibited consistently higher diversity and evenness over time, reaching a peak in 2021. During this period, both shrub and herbaceous layers supported a greater number of species compared to the other treatments, with species richness increasing to varying extents and showing a clear upward trend during the early recovery phase. This suggests that the canopy gaps created by the 1/2 harvesting intensity provided optimal light, temperature, and water conditions—sufficient to prevent dominant species from monopolizing resources while avoiding harsh environmental extremes. As a result, understory vegetation could regenerate rapidly, enhancing plant diversity and promoting ecosystem stability. These findings are consistent with the Intermediate Disturbance Hypothesis and the Maximum Disturbance Threshold theory [9,23,45,46,47], and are in agreement with the results of Zhan Meichun et al. Moreover, in the fifth year, species diversity under the clear-cutting treatment was also found to be higher than under selective harvesting. This may be attributed to the greater disturbance intensity associated with clear-cutting, which leads to higher nutrient loss, lower stand density, and reduced canopy closure, thereby allowing for more stable water and light availability and ultimately higher species richness. However, as time progresses, species richness in such treatments is likely to decline. Areas with lower species richness are more susceptible to invasion by alien species, and their plant communities tend to be more easily disturbed and structurally unstable [48]. Both one-way and two-way ANOVA revealed significant differences in diversity indices across different recovery years under the same treatment, indicating that temporal factors play a critical role in community recovery and successional dynamics. During the five-year post-harvest recovery period, understory plant diversity in bamboo forests demonstrated a dynamic trajectory in response to clump harvesting. These results are consistent with previous research findings [49,50,51,52]. In summary, while species richness remained stable by the fifth year, the decline in diversity indices indicates that the community structure is beginning to shift toward homogenization. The findings support the Intermediate Disturbance Hypothesis, suggesting that moderate levels of stand disturbance can similarly maximize species diversity within bamboo forest ecosystems. In forest management practices oriented toward economic objectives, regulating harvesting intensity offers an effective strategy to shape the community structure and enhance biodiversity in sympodial bamboo forests, thereby promoting a balanced achievement of both ecological and production goals.

### 4.3. Relationships Between Soil Chemical Properties, Stand Structure, and Species Diversity

Climatic microenvironments created by harvesting drive the distribution of plant communities [53]. Plant diversity is influenced by multiple environmental factors, with large-scale patterns shaped by elevation and climatic conditions, and small-scale variations largely determined by topography and soil factors [54]. As the material foundation for plant survival, soil properties are strongly influenced by stand spatial structure, which affects the composition of understory plant communities, soil texture, and litter characteristics. These differences in turn indirectly affect soil nutrient status [55]. In this study, both understory plant diversity and soil chemical properties showed notable changes during the recovery period after harvesting in *D. giganteus* stands. Principal component analysis indicated a significant correlation (*p* < 0.05) between diversity and soil chemistry in the early recovery stage (years 1–3), while no significant correlation was found in Year 5 (*p* > 0.05) [56], suggesting that the direct influence of soil factors on diversity is time-dependent [57]. This may be due to early post-harvest disturbances altering the forest structure and understory resource availability. Increased surface soil fertility and expanded canopy gaps allowed herbaceous and shrub species to thrive and regenerate, resulting in rising diversity indices. In contrast, in the later recovery stage, reduced light availability likely became the main limiting factor. As canopy density increased, light penetration to the understory declined, suppressing herbaceous growth and reducing diversity. Dominant species such as *A. hispidus* and *C. patens* were gradually replaced, and community structure tended to simplify. This trend aligns with the findings of Shuweiwei et al. [58,59], who identified light availability as a key driver of diversity patterns. Additionally, *D. giganteus* is a fast-growing clonal bamboo, and its understory composition responds more sensitively to disturbance. In the early recovery stage, large amounts of litter decomposition and root turnover contributed to rapid organic matter release, enhancing microbial activity and soil carbon accumulation [60], thereby promoting herbaceous expansion [55,61,62]. As nutrients were depleted and litter input declined, surface soil fertility decreased. Since shallow-rooted herbaceous species rely heavily on topsoil nutrients, they eventually declined due to organic matter limitations. In summary, plant diversity in the early post-harvest period is jointly driven by soil nutrients and light conditions, while light becomes the dominant limiting factor in later stages. The interplay between soil and light significantly shapes the spatiotemporal dynamics of understory biodiversity.

## 5. Conclusions

This study demonstrates that clump-based harvesting can effectively enhance the species’ richness, abundance, and diversity indices of understory vegetation. The species diversity of understory plants exhibited a dynamic trend of first increasing and then decreasing over the restoration period, and the plant community structure gradually shifted toward simplification. These findings indicate that the positive effects of clump-based harvesting on understory biodiversity are time-limited and not sustained in the long term. The removal of *D. giganteus* biomass following harvesting led to changes in the soil chemical properties, which in turn affected plant growth and community diversity. Significant correlations were observed between soil chemical properties and species diversity in the early stages of harvesting (Year 1, *p* < 0.05), whereas no significant correlations were detected by Year 5 (*p* > 0.05). Based on fuzzy membership function analysis, the 1/2 clump-based harvesting treatment exhibited the highest overall performance in terms of understory species diversity, with average values significantly higher than those of the other treatments. This suggests that the 1/2 harvesting strategy is most conducive to the healthy development of *D. giganteus* stands. Therefore, to determine the optimal management and cultivation model for sympodial bamboo forests, it is essential to conduct a comprehensive assessment of various harvesting intensities from ecological, economic, and temporal perspectives. Such an approach will enable the formulation of scientifically grounded clump-based harvesting strategies, thereby supporting the sustainable management of *D. giganteus* forests.

## Figures and Tables

**Table 1:** Basic characteristics of the experimental sites.

Treatment	2018 Before Harvesting		2018 After Harvesting		2021		2024	
	Density (Stems·ha^−1^)	Mean DBH (cm)	Density (Stems·ha^−1^)	Mean DBH (cm)	Density (Stems·ha^−1^)	Mean DBH (cm)	Density (Stems·ha^−1^)	Mean DBH (cm)
A (Selective cutting)	2970	12.87	1782	11.92	3371	12.8	3584	10.27
B (1/2 clump cutting)	2939	11.80	1959	10.81	3760	12.62	4025	11.36
C (1/3 clump cutting)	3024	12.19	1512	10.96	1766	8.49	2158	10.19
D (Whole clump cutting)	2904	11.46	-	-	2279	5.93	2434	8.76

**Table 2:** Regression models of aboveground biomass for *D. giganteus* at different age classes.

Age Class	Regression Model Ba = a·DBH^b^	Applicable Age Range
Class I	Ba = 0.089·DBH^2.378^	≤1 year
Class II	Ba = 0.1751·DBH^2.4262^	1–3 years
Class III	Ba = 0.2305·DBH^2.3255^	>3 years
All ages	Ba = 0.1889·DBH^2.405^	All age classes

Ba = aboveground biomass (kg/plant); DBH = diameter at breast height (cm).

**Table 3:** Two-way ANOVA results for the effects of harvesting intensity and recovery time on understory plant diversity indices.

Indicator	Recovery Time			Logging Intensity			Logging Intensity × Recovery Time		
	df	F	*p*	df	F	*p*	df	F	*p*
Simpson index	2.00	58.95	0.00	3.00	38.42	0.00	6.00	12.11	0.00
Margalef index	2.00	3303.21	0.00	3.00	2376.45	0.00	6.00	591.52	0.00
Shannon–Wiener index	2.00	1198.38	0.00	3.00	576.00	0.00	6.00	91.93	0.00
Pielou index	2.00	279.32	0.00	3.00	137.50	0.00	6.00	13.56	0.00

**Table 4:** Comprehensive evaluation of diversity indices, stand characteristics, and soil chemical properties based on fuzzy membership function analysis.

Treatment	2019				2024			
	A	B	C	D	A	B	C	D
pH	0.81	1.00	0.43	0.00	0.72	1.00	0.93	0.00
SOC	1.00	0.94	0.89	0.00	1.00	0.68	0.11	0.00
AN	0.62	1.00	0.43	0.00	1.00	0.60	0.58	0.00
AP	0.00	0.70	1.00	0.37	0.00	1.00	0.60	0.16
AK	0.55	0.00	1.00	0.33	1.00	0.00	0.13	0.40
TN	0.60	1.00	0.65	0.00	0.00	1.00	0.38	0.17
TP	0.60	0.81	1.00	0.00	1.00	0.85	0.90	0.00
TK	0.87	1.00	0.69	0.00	0.39	0.94	1.00	0.00
Shannon–Wiener index	0.78	1.00	0.42	0.00	0.00	1.00	0.58	0.49
Simpson index	0.92	1.00	0.63	0.00	0.00	0.73	1.00	0.49
Pielou index	0.55	1.00	0.34	0.00	0.00	1.00	0.41	0.47
Margalef index	0.65	1.00	0.41	0.00	0.00	1.00	0.67	0.37
Stand density	0.55	1.00	0.00	0.30	0.76	1.00	0.00	0.15
Diameter at breast height	0.61	0.40	1.00	0.00	0.58	1.00	0.55	0.00
Biomass of individual plant	0.54	0.32	1.00	0.00	0.57	1.00	0.52	0.00
Average	0.64	0.81	0.66	0.07	0.47	0.86	0.56	0.19
Rank	3	1	2	4	3	1	2	4

## Data Availability

Data is contained within the article.
